# Complete Resolution of Skull Base Solitary Plasmacytoma Using Proton-Beam Radiotherapy: A Case Report

**DOI:** 10.7759/cureus.23130

**Published:** 2022-03-13

**Authors:** Hamza Khilji, Caroline Silver, Doaa Morrar, Arpit M Chhabra, Steven Mandel, David J Langer, Dana Shani, Jason A Ellis

**Affiliations:** 1 Neurosurgery, Lenox Hill Hospital/Donald and Barbara Zucker School of Medicine at Hofstra/Northwell, New York, USA; 2 Hematology-Oncology, Lenox Hill Hospital/Donald and Barbara Zucker School of Medicine at Hofstra/Northwell, New York, USA; 3 Pathology, Lenox Hill Hospital/Donald and Barbara Zucker School of Medicine at Hofstra/Northwell, New York, USA; 4 Radiation Oncology, New York Proton Center, New York, USA; 5 Neurology, Lenox Hill Hospital/Donald and Barbara Zucker School of Medicine at Hofstra/Northwell, New York, USA

**Keywords:** skull base, radiotherapy, proton radiation, multiple myeloma, chemotherapy, brain tumor

## Abstract

Cranial solitary plasmacytomas are uncommon lesions, and localization to the skull base is rare. Here we present a case in a 36-year-old woman who complained of dizziness and mild headaches. Radiographic imaging indicated the presence of a solitary skull base lesion in the posterior cranial fossa. Laboratory tests and imaging excluded systemic disease. A biopsy of the lesion confirmed the diagnosis of plasmacytoma. The patient was treated with proton-beam radiation and had a complete clinical and radiographic resolution, demonstrating the previously unreported utility of monotherapy with proton-beam radiation in such cases.

## Introduction

Plasmacytomas arise from an abnormal proliferation of B-cell lymphocytes within the bone or soft tissue. Although rare, plasmacytomas of the skull base have previously been reported. The radiographic differential diagnosis for such tumors at the skull base is wide, but the more common pathologies include meningioma, chordoma, osteosarcoma, metastasis, and others [1]. Classic symptoms of skull base plasmacytomas include headache, double vision [2], and vertigo [3].

Specific categorization of plasmacytomas is based on histologic, anatomic, radiographic, and clinical criteria [4,5]. Presentation of a singular mass is denoted as “solitary,” while systemic involvement may indicate multiple myeloma (MM). Solitary plasmacytomas (SP) are further sub-classified based on location in bone (intramedullary) or soft tissue (extramedullary). Patients with solitary intramedullary plasmacytoma (SIP) have a <30% chance of progressing to multiple myeloma, while those with solitary extramedullary plasmacytoma (SEP) have a > 50% chance of progression [6].

The diagnostic workup for a solitary plasmacytoma includes a biopsy with evidence of clonal plasma cells, radiographic images demonstrating a lack of systemic lytic lesions, and a bone marrow aspirate and biopsy with no clonal plasma cell presence [7]. Whole-body computed tomography (CT) or positron emission tomography (PET) imaging are the preferred diagnostic imaging modalities [8].

When a solitary plasmacytoma is suspected, MM, POEMS syndrome, and metastatic carcinoma should be ruled out. Patients presenting with 10% or greater abnormal plasma cells in the bone marrow, systemic lesions, and renal insufficiency, likely have a diagnosis of MM. Patients with POEMS syndrome (Polyneuropathy, Organomegaly, Endocrinopathy, Monoclonal protein, Skin changes) present with a singular osteolytic bone lesion, defined by a sclerotic rim, a small quantity of monoclonal paraprotein, and elevated levels of vascular endothelial growth factor [9]. Patients with metastatic carcinoma present with multiple lytic lesions and <10% clonal plasma cells in the bone marrow; however, this may be more closely associated with an unrelated monoclonal gammopathy, rather than an SP, distinguishable by a lesion biopsy [7]. 

Since plasmacytomas are highly chemo/radio-sensitive than several other skull base tumors, prompt and accurate diagnosis is imperative [1,3,10]. Here we present a unique-and to our knowledge, previously unreported-case of a skull base plasmacytoma treated solely with proton radiation therapy. The patient demonstrated an excellent radiographic and clinical response.

## Case presentation

A previously healthy 36-year-old woman on no medications presented with dizziness and mild headaches. The physical and neurological examination was unremarkable.

**Figure 1:**
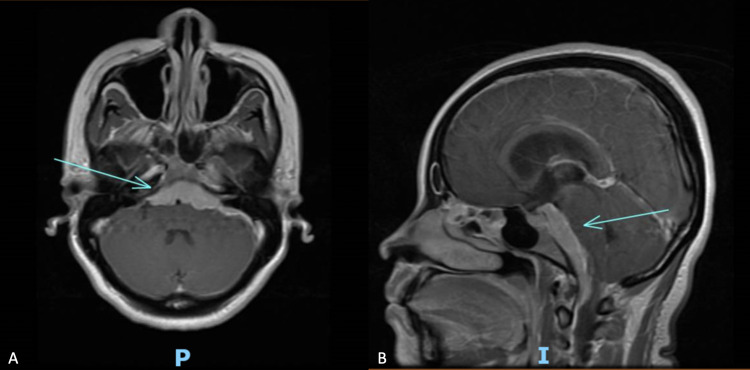
Pre-treatment brain MRI with contrast in axial (A) and sagittal (B) views demonstrate bulky enhancing tumor ventral to the pons at the skull base.

Magnetic resonance imaging (MRI) and CT scans revealed an enhancing posterior cranial fossa skull base tumor with bulky disease anterior to the brainstem and medulla, extending into the left cerebellopontine angle (Figure 1). Due to the wide differential diagnosis, including en plaque meningioma, the patient was taken for a left retrosigmoid craniotomy to biopsy the tumor.

**Figure 2:**
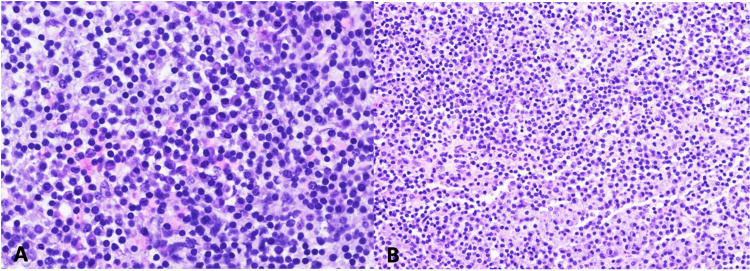
Microscopic examination shows a highly cellular tumor. The tumor comprises sheets of plasma cells and scattered lymphocytes which can be seen here at both high (A) and low magnification (B). The plasma cells have a moderate amount of cytoplasm. Mitoses were identified, and hemorrhagic areas were seen between the tumor cells.

**Figure 3:**
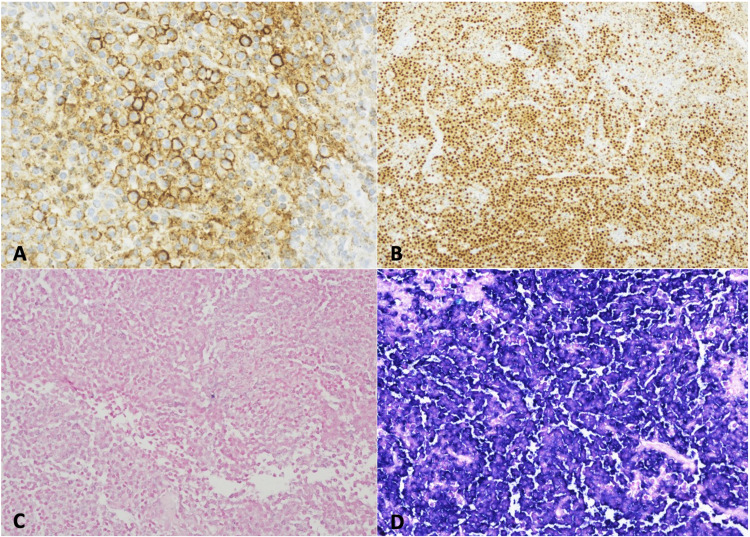
Immunohistochemistry showed the tumor to be CD 138 positive (A), MUM-1 positive (B), kappa negative (C), and lambda-positive (D).

Immunohistochemical studies demonstrated positive staining of the plasma cells for CD138 and MUM-1 and negative staining for cyclin D1. A stain for CD20 highlighted B-lymphocytes in the background, and in situ hybridization studies for kappa and lambda showed lambda light chain restriction in the plasma cells. While the initial frozen section evaluation suggested possible lymphoma, the final specimen analysis was consistent with a plasma cell neoplasm (Figure 2, 3).

A complete blood cell count with differential revealed iron deficiency anemia, and serum creatinine revealed normal kidney function. Immunoelectrophoresis revealed the presence of an IgG lambda monoclonal paraprotein, suggesting possible MM. Additional laboratory and radiographic tests were done to rule out MM and systemic disease (Table 1).

**Table 1:** Diagnostic Testing PET-CT-FDG: fluorodeoxyglucose-positron emission tomography

Type of Test	Pertinent Finding(s)	Reference Value
CBC with differential	RBC Count: 3.70 M/uL	3.80-5.20 M/uL
	HGB: 9.7 g/dL	11.5-15.5 g/dL
Serum β2 microglobulin	1.1 mg/L	0.8-2.2 mg/L
Metabolic Panel	Creatinine: 0.56 mg/dL	0.5-1.30 mg/dL
	Albumin: 4.4 g/dL	3.3-5.0 g/dL
Serum Immunoelectrophoresis	Beta-migrating paraprotein identified	n/a
	M-spike: 0.2 g/dL	0.0-0.0 g/dL
Serum Immunofixation	IgG lambda monoclonal paraprotein Identified: 1562 mg/dL	610-1660 mg/dL
Serum Ferritin	30 ng/ml	15-150 ng/ml
Bone Marrow Aspirate and Biopsy	No evidence of plasma cell myeloma	n/a
PET-CT FDG Skull to Thigh	No FDG-avid disease	n/a
Flow Cytometry	Insufficient for evaluation due to low specimen viability	n/a
Tumor Biopsy	Lambda light chain restricted neoplasm	n/a

The serum β2 microglobulin, albumin, and ferritin levels were normal. A whole-body PET-CT indicated no 2-(18F) fluoro-D-glucose (FDG) avid disease. A CT-guided bone marrow aspirate and biopsy confirmed a lack of systemic involvement, thereby excluding a diagnosis of MM.

**Figure 4:**
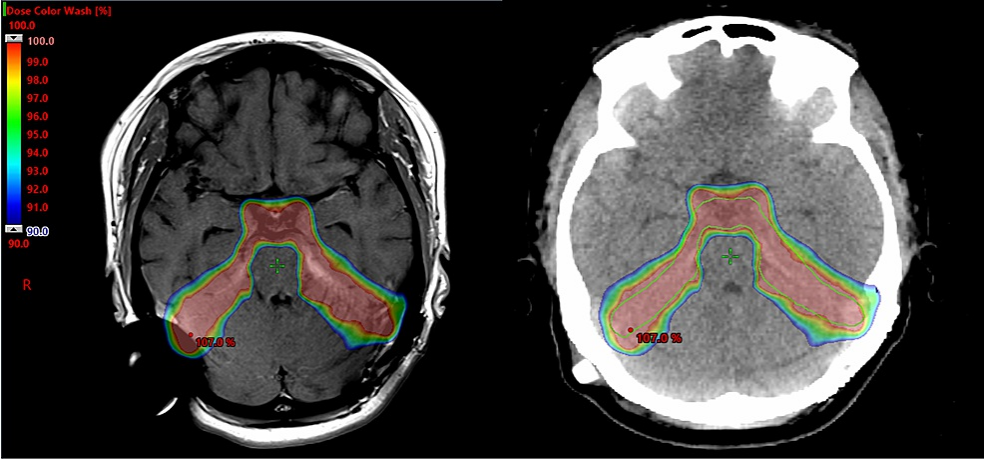
Proton-beam treatment plan. Here the dose spectrum highlights the steep radiation dose fall-off with limited exposure to the surrounding brain tissue. The target prescription dose of 45Gy (100%) is shown down to 40.8Gy (90% of prescription dose).

**Figure 5:**
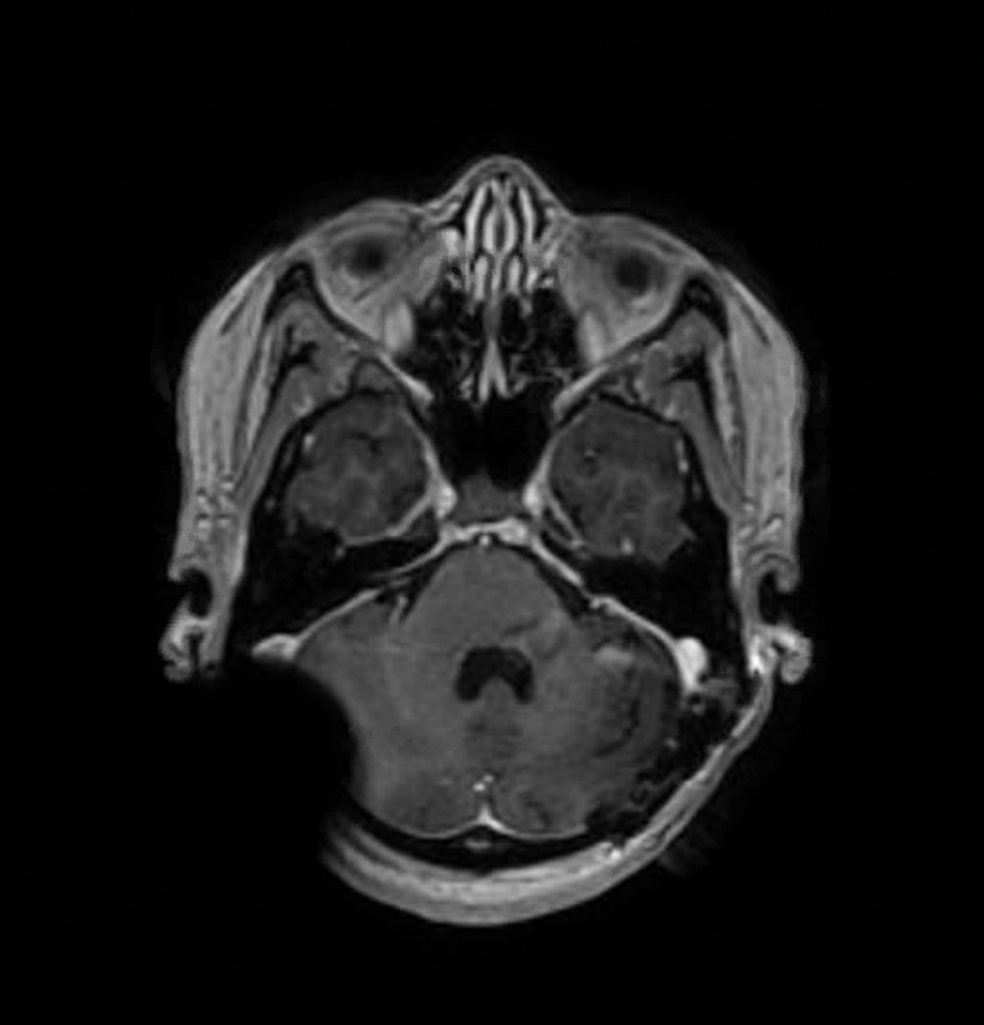
Contrast-enhanced MRI after completion of proton-beam radiotherapy shows complete resolution of the tumor.

The decision was then made to initiate treatment with proton beam radiotherapy using a 45 Gy dose delivered over 25 fractions (Figure 4). Follow-up imaging demonstrated complete resolution of the tumor (Figure 5). At three months follow-up, the patient remained asymptomatic, and the lambda monoclonal paraprotein was no longer detected.

## Discussion

Solitary plasmacytomas (SP) comprise <10% of plasma cell neoplasms, while those isolated to the skull base are even rarer [1,3]. Nonetheless, patients with osseous plasmacytomas are at high risk for developing multiple myeloma (MM) in the years after treatment and require long-term monitoring [11]. While solitary plasmacytomas can progress to MM, they do not present with the typical clinical manifestations of MM, such as anemia and renal failure [12]. Thus, MM should always be initially ruled out in a patient with suspected SP not wrongfully to withhold needed systemic treatment [10,13].

We have found 12 previous reports of skull base SP identified in the literature from 2000 to 2018 (Table 2). The mean age of the patients was 49.2 years (range 28 to 66) at the time of diagnosis, and no gender predilection was noted. Anatomically, tumor locations include six cases in the clivus, one in the sphenoid sinus, one in the cavernous sinus, and four with spheno-clival involvement. The most common presenting symptoms were headache and diplopia, attributable to associated third and sixth cranial nerve involvement [1,2,14]. The most common treatment reported was surgery followed by conventional radiotherapy (RT). The role of surgery in treating SPs is limited to diagnostic purposes and to assist with tumor debulking in lesions causing mass effect and neurologic deficits [1,2]. Conventional RT with a 40-50 Gy dose is typical [11,15,16].

**Table 2:** Solitary Plasmacytomas of the Skull Base F, Female; M, Male; GTR, Gross Total Resection; STR, Sub-Total Resection; PR, Partial Resection; CT, Chemotherapy; RT, Radiotherapy

Case	Date	Age	Sex	Location	Symptoms	Treatment	Radiation Dose	Follow-Up (months)	Author
1	2018	41	M	Central skullbase, clivus	Headache, diplopia, left eye strabismus	GTR, RT	50 Gy/25	3, stable	Siyag et al.
2	2012	50	F	Middle-upper clivus	Headache, diplopia	GTR, RT	46,8 Gy/26	165, stable	Gagliardi et al.
3	2012	53	F	Middle-upper clivus	Diplopia	GTR, RT	40 Gy/22	9, stable	Gagliardi et al.
4	2012	57	M	Upper clivus	Headache	STR, RT	45 Gy/25	20, stable	Gagliardi et al.
5	2012	66	F	Sellar region, upper clivus and sphenoid sinus	Bitemporal headache, diplopia	GTR, patient denial of CT and RT	n/a	3, death	Guinto-Balanzar et al.
6	2010	40	M	Clivus	Headache, blurry vision, diplopia,	RT, Thalidomide, Dexamethasone	30 Gy/10	2, stable	Kashyap et al.
7	2009	32	M	Sphenoid sinus	Ocular pain, diplopia	PR, RT	4,000cGy/20	8, stable	Park et al.
8	2008	54	F	Spheno-clival	Headache, right eye hemianopia, bilateral blind spot enlargement	PR, RT	45 Gy	22, stable	Liu et al.
9	2007	58	F	Right anterior petrous apex and clivus	Headache, right facial numbness	RT	45 Gy	18, stable	Husein et al.
10	2003	61	F	Cavernous sinus	Deteriorating vision, headaches	PR, RT	50 Gy	12, mass resolution	Brannan et al.
11	2003	50	M	Clivus	Binocular diplopia, headaches	RT, CT	45 Gy/25	8, stable	Brannan et al.
12	2000	28	M	Sphenoclival	Bifrontal headaches, diplopia	RT	5,400 cGy	3.5, stable	Wein et al.

In the patient we present, proton radiotherapy resulted in complete tumor resolution within one month. Although the efficacy of proton beam therapy for myeloma and plasmacytoma has been suggested due to their known radiosensitivity, no rigorous studies have been performed [17,18]. This report is the first case demonstrating the utility of proton beam radiotherapy for a tumor of this kind in the skull base. The sharp dose fall-off profile of proton radiation makes it more appealing to use in the skull base than conventional RT [19]. This unique feature of proton beam therapy is especially valued in young patients.

As previously mentioned, SPs can progress to MM; however, the use of systemic therapy such as VRD (Velcade, Revlimid, and decadron) [20] is not thought to be preventative. Therefore, continued vigilant follow-up is indicated for patients like our present [1,21-24].

## Conclusions

The case presented demonstrates the potential clinical utility of proton-beam radiation therapy as monotherapy in patients with skull base solitary plasmacytoma. In clinical scenarios such as this, consideration should be given to such a treatment approach to limit adjacent tissue radiation adverse effects and systemic toxicity. Additional studies will be needed to demonstrate the generalizability of this technique.
